# Sustainability of food production support services offered by Sustainable Agriculture Trust to subsistence farmers in Bikita District, Zimbabwe

**DOI:** 10.4102/jamba.v11i1.526

**Published:** 2019-05-20

**Authors:** Norman Chivasa

**Affiliations:** 1 International Centre of Non-violence, Peacebuilding programme, Durban University of Technology, Durban, South Africa; 2 Department of History, Unit of War and Strategic Studies, University of Zimbabwe, Harare, Zimbabwe

## Abstract

In developing countries, food production challenges continue to persist, despite interventions at household levels such as input provision to address farmers’ resource constraints and training in conservation farming to improve land use and management by farmers. In Bikita District of Zimbabwe, concerns over the viability of conservation farming and input support services in helping out subsistence farmers to mitigate food production challenges remain unresolved. This research, therefore, evaluated the sustainability of support services offered by the Sustainable Agriculture Trust to subsistence farmers taking Ward 13 of Bikita District as a case study. Data collection involved two qualitative methods, namely structured interviews and focus group discussions, with 32 household heads comprising 18 women and 14 men. Results showed that the capacity by subsistence farmers to continue practising conservation farming using retained maize, sorghum and cowpea seed varieties and still produce better yields even after the Sustainable Agriculture Trust’s exit justify the hypothesis that conservation farming is a sustainable food production intervention. This study therefore concludes that the proficiency by subsistence farmers in Ward 13 of Bikita District carries the implication that training household heads in conservation farming means developing more subsistence farmers capable of producing their own food even amidst unpredictable rains, heat waves and shortening rainfall seasons.

**Keywords:**

food production; food security; subsistence farming; sustainability; conventional farming.

## Introduction

Globally, statistics on the number of people experiencing food shortages, starvation, hunger and malnutrition in developing countries continues to be on the increase to unprecedented levels. For example, the Food and Agriculture Organization (FAO) (2004), cited in Abu and Soom (2016:56), estimated that 800 million people were experiencing food crisis in 2004. The FAO (2010) cited in Sekhampu (2013:543) estimated that figure was 900 million. The FAO (2012) cited in Zakari, Ying and Song (2014:1192) estimated 852 million. These statistics point to the scale of the food crisis facing the developing world, and Zimbabwe has not been spared either.

Arguably, Zimbabweans are currently faced with erratic income streams and food insecurity problems because of instability in the economic sector and other factors (Nyakwawa 2015:3). FinScope Zimbabwe (2011:2) reported that about 61% of Zimbabweans go without cash at some stage while 36% skip a meal because of a lack of money or food. Fowler and Panetta (2010:2) noted that agriculture, which forms the basis of food production, especially for the majority of rural people in Zimbabwe, has declined sharply because of persistent droughts and economic crises over the past decades, affecting food production. From their perspective, decline in food production with attendant poverty and hunger has propelled some rural people to devise alternative livelihood strategies. For example, Gukurume (2013:89) examined climate adaptation and mitigation strategies in the face of crop failure and low yields (declining food production) by subsistence farmers in Bikita District. He found that subsistence farmers have responded to poverty and hunger by adopting conservation farming (CF) and crop diversification.

Poverty in rural wards in Zimbabwe is more widespread than in urban wards. For example, statistics indicate that the poverty prevalence rate in rural wards stands at 85%, while the poverty prevalence in urban wards is at 37.2% (United Nations Children’s Fund [UNICEF] 2015:xiii). A ward is an administrative unit with membership drawn from villages (Kurebwa 2015:104), and it is under the leadership of a politically elected councillor. A group of wards constitute a district.

Bikita District, which is the focus of this study, is one of the districts that have not been spared by the scourge of poverty. It is one of the seven districts in Masvingo Province, which lies along the Masvingo–Mutare Highway. It falls into the medium drought risk zone in which maize is the main crop produced by subsistence farmers (Chikobvu et al. 2010:1).

Bikita District has 31 wards. For some time it has been acknowledged that levels of livelihood insecurity among subsistence farmers in Bikita District are high and that a significant part of such insecurity involves food insecurity. Food insecurity in Bikita District occurs within the context of several factors such as crop failure (Matthew 2003:140); unpredictable rains, heat waves, shortening rainfall seasons (Gukurume 2013:89); poorly distributed rainfall (Mushore, Mudavanhu & Makovere 2013:102); gross incidence of drought (Chitongo 2013:53); declining soil fertility and attendant declining food production (Mapuranga et al. 2015:1); inadequate institutional support; poor harvests (Fowler & Panetta 2010:2; UNICEF 2016) and liquidity challenges (Famine Early Warning Systems Network [FEWSN] 2016:4).

Bikita District constitutes one of the most populous districts in Masvingo Province, with the primary livelihood being crop production of maize, sorghum, millet and groundnuts. A report by the Zimbabwe National Statistics Agency (2015:186) showed that Bikita District has a 72.1% poverty prevalence rate. Households there normally rear cattle, chickens, goats and donkeys but still have poor consumption and dietary diversity patterns. As a result, certain households exchange labour for vegetables while others have resorted to small-scale trading to supplement their dietary requirements. Overall, households in Bikita District consume cereals and vegetables twice a day (FEWSN 2016:7, 8).

To supplement the food production capacities of subsistence farmers in Bikita District, four non-governmental organisations (NGOs) – namely, Action Aid, CARE International, Foundations for Farming and Sustainable Agricultural Trust (SAT) – have provided support services in the form of training in CF as well as inputs and other extension services (Mafongoya n.d.:3). This article was set to evaluate the sustainability of food production support services offered to subsistence farmers by SAT in Ward 13 in Bikita District. The central research problem faced by this study may be stated as follows:

- Can support services offered by SAT promote sustainable food production capacities of subsistence farmers in Ward 13 in Bikita District even when beneficiaries seem inimical to training in CF, which is an essential precondition for improved household food production capacities?

Sustainable Agricultural Trust’s (SATs) CF intervention has five major methods. The first is training in the technique of planting in holes or basins in the planting area. The second is training in the application of pesticides to deter pests that negatively affect crops. The third method is the provision of extension services, training in weed control and residual moisture management. The fourth method is training farmers to increase food production capacities to minimise donor dependence; this training involves technical details such as in-row spacing for maize seed, beans, sorghum and the application of fertiliser. Finally, the exit strategy for SAT is that subsistence farmers are trained for 2 years for potential proficiency in crop production (FAO 2008:4).

In theory, it can hardly be disputed that the benefits arising from the CF initiative have a long-term impact on the lives of the beneficiaries. These benefits are not only limited to socio-economic prosperity of subsistence farmers but include improved land use and soil fertility, which leads to improved crop yields (Mapuranga et al. 2015:6). It follows that training in CF adds value to input support services by incorporating land use and management. Accordingly, input constraints and soil infertility problems are compensated for through SAT’s support services to subsistence farmers (FAO 2008:2).

Ironically, a few studies that have specifically focused on the adoption of CF by subsistence farmers in Bikita District have produced mixed results. Gukurume (2013:89) examined climate adaptation and mitigation strategies in the face of crop failure and low yields (declining food production) by subsistence farmers in Bikita District. He found that subsistence farmers had responded by adopting CF and crop diversification. In contrast, Chitongo (2013:53) assessed inherent contestations that militate against the effectiveness of CF in Bikita District as a food insecurity mitigatory measure in Ward 8. He found that CF had not done much to mitigate food insecurity challenges because it was imposed by NGOs and it did not build on the local knowledge of the people in Bikita District.

Similarly, studies by Mafongoya (n.d.:3) found that CF was received with mixed reactions in Ward 21 of Bikita District. He found that subsistence farmers considered the use of tools such as hoes or shovels, digging of planting basins and application of compost manure – which are dominant in CF practice –to be ‘[l]abor intensive and sometimes contradicting their orthodox farming techniques’ (Mafongoya n.d.:3). As a result, relations between SAT and CARE International officials became frosty as beneficiaries resisted the adoption of CF (p.8).

While Chitongo’s and Mafongoya’s findings raised concerns about the viability of CF in helping farmers, food production capacities and other scholars found CF useful in improving the livelihoods of subsistence farmers. For example, Mapuranga et al. (2015:5) appraised the contribution of CF towards household food security in Bikita District. They found that CF had greatly contributed to improved food production at household level in the district. Owing to these merits, Mapuranga et al. (2015:6) recommended that all communal areas in Zimbabwe consider adopting CF. These results refute the notion that interventions follow a linear path that commences with training in CF and provision of inputs, moves to proficiency and ends with improved food production capacity of subsistence farmers. These mixed results over the viability of CF in helping subsistence farmers’ food production capacities in Wards 8 and 21 of Bikita District was the impetus behind this article to explore issues of sustainability in food production support services. Note that the term ‘sustainable’ in relation to food production only came into use when the Zimbabwean government adopted conservation agriculture (CA) as a farming system in the 1990s, and NGOs subsequently adopted and promoted CF in rural areas in the early 2000s (more details on CA and CF are provided under the literature review section) (Harford, Le Breton & Oldreive 2009:3; Marongwe et al. 2012:9). Hence, the use of the term in this article.

Hanyani-Mlambo et al. (2016) define ‘sustainability’ as:

The extent to which the project has established and built institutional capacity that ensures the continuation and maintenance of the project outcomes after the period of external support has ended. (p. 16)

This study evaluates the sustainability of support services offered by SAT to subsistence farmers in Ward 13 of Bikita District. Ward 13 is one of the low-rainfall, high-temperature wards in Bikita District (Sustainable Agriculture Technology [SAT] 2014).

Consequently, to address the central research problem of this study, three objectives were put forward:

- to examine farming methods used to grow crops by subsistence farmers
- to assess the nature and suitability of SAT support services offered to subsistence farmers
- to explore whether subsistence farmers would become proficient in crop production after SAT had left.

To address the central research problem systematically, the study is structured as follows. Firstly, literature relating to the food production framework is examined. Next, the setting and research design are described, as well as data collection methods and ethical considerations. Practical implications of the study are then addressed, followed by the study conclusion.

### Literature review

#### Conservation agriculture as a framework for food production

Conservation agriculture is a farming technology that was associated with large-scale commercial farming in North America, Brazil, Australia, Argentina, China, India, Europe, Morocco, South Africa and Paraguay in the 1930s (Wagstaff & Harty 2010:68). It is built around three major principles. The first is reduction in soil disturbance. The second is maintenance of soil cover and the third is crop rotation (Harford et al. 2009:3; Marongwe et al. 2012:3; Wagstaff & Harty 2010:69–70).

In a world increasingly faced with decline in food production, CA farming technology has taken centre stage in addressing the food production capacity problem across the globe (Giller et al. 2009; Marongwe et al. 2012:3). Although in the literature, ‘food production’ relates to both crops and livestock production as the major source of food (i.e. both cereal and livestock products) for every household, for the purposes of this study ‘food production’ means production of staple crops, namely maize, sorghum, millet, rapoko and wheat, just to mention a few. This is true of subsistence farmers in sub-Saharan Africa (SSA), in particular, in Malawi, Mozambique and Zimbabwe, who focus on producing grain (Chisoko 2003:3). This study is interested in the crop production capacities of subsistence farmers.

Harford et al. (2009:9) noted that since the early 1960s, SSA has experienced a sharp decline in cereal production. Major factors that account for this decline include but are not limited to poor land use management, mismanagement of water resources, erratic rainfall, insufficient land to accommodate the growing population and livestock, just to mention a few. By implication, a farming system with the capacity to improve land use and water management becomes critical. Marongwe et al. (2012:3) singled out CA as having remedial capacities to address the decline in soil fertility and mitigate the problems of erratic rainfall and reduced food production.

The long-term benefits of using CA even among problems such as reduced food production, destroyed soil fertility and erratic rainfall are enormous. For example, Wagstaff and Harty (2010:70) note that CA prevents soil infertility because of its capacity to minimise soil erosion and increase soil organic matter. Furthermore, they maintained that CA is accountable for improved rainwater infiltration and reduction in the evaporation of water from the soil. According to them, the decrease in water evaporation from the soil implies that the planting basins have high water-holding capacity that enables plants to reach maturity (p. 70). Harford et al. (2009:3) contend that the practice of CA increases food production while at the same time preserving natural resources such as land and organisms that live in the soil. They argued that land is preserved because through CA, the destruction of soil structure is reduced and, in that way, soil is not exposed to the wind and soil erosion. Similarly, organisms that live in the soil are minimally disturbed, thereby increasing soil organic matter because of the practice of reduced tillage in CA (p. 3). Among the short-term benefits of CA to subsistence farmers, Harford et al. (2009:13) quoted inputs such as seeds and inorganic fertiliser, which are spot-placed where needed. As a result, although there is less use of inputs and fertiliser or manure, these smaller amounts used efficiently have the potential to produce higher yields.

A useful historical perspective on CA in Zimbabwe is necessary to enable an assessment of the scale and scope of this farming system, the problems it has faced and must also address for the future. Historically, Zimbabwe adopted three faming systems: CA, CF and ConvT (Conventional Tillage). To avoid conceptual discord these farming systems are clarified. Conservation agriculture is defined by Mvumi, Ndoro and Manyiwo (2017) as:

… a broader term that encompasses activities such as minimum tillage and zero tillage, tractor powered, animal powered, manual methods, integrated pest management, integrated soil and water management, and includes CF. (p. 1630)

Central to CA is the use of ox-drawn or other mechanised equipment (ripper tines, direct seeders or jab-planters). Less soil tillage is central to CA (Harford et al. 2009:3). In contrast, Mvumi et al. (2017:1630) define CF ‘as digging planting basins using hoes, following principles like mulching and crop rotation’. Conservation farming primarily uses hand-hoe basins, which explains why it is popularly called the ‘planting basin technique’ in Zimbabwe (Harford et al. 2009:3).

Conventional Tillage, on the other hand, uses the ‘[*m*]old-plough in land preparation for the whole area to be planted and then planting is done following behind the plough’ (Mvumi et al. 2017:1630). Conventional Tillage uses a moldboard ox-drawn plough for ploughing. As a result, ConvT loosens the soil to a suitable depth where plant roots can easily penetrate. Soil tillage is central to ConvT (Harford et al. 2009:10). The difference between these three farming practices lies with the type of equipment used when planting seed and the attendant effects on land. Of the three farming methods, ConvT is notorious for causing destruction of soil structure, leading to poor crop yields because of soil infertility and increasing environmental degradation (Harford et al. 2009:10).

Ironically, a study by Mvumi et al. (2017:1634) on farming practices in Zimbabwe showed ConvT as the top prioritised farming practice of the three. According to them, ConvT is adopted because most farmers inherited this farming system from their forefathers, which explains why it is called the ‘traditional farming system’. The second best is CF, which is a transformed version of CA (Harford et al. 2009:3). Thomlow (2007) cited in Mvumi et al. (2017:1630) and Harford et al. (2009:3) contended that in post-independence Zimbabwe, CF was adopted and integrated into the ConvT system. Mvumi et al. (2017:1634) cited the lack of mechanised equipment by subsistence farmers as the major impetus behind the adoption of CF.

According to Marongwe et al. (2012:3), an estimated 70% of the Zimbabwean population depends on agriculture, while 75% depends on rain-fed farming. They indicate that Zimbabwe is faced with declining soil fertility, erratic rainfall and high input costs, all of which account for the food production problems. In the same report, it was pointed out that the problems of increased land degradation, soil fertility decline and high input costs go back to the 1920s in colonial Zimbabwe. As a result, after independence in 1980, an estimated 30% of commercial farmers believed to have been experiencing land degradation and soil fertility problems adopted the CA to remedy these problems (Marongwe et al. 2012:8).

In spite of these mitigatory measures, Zimbabwe experienced a steady decline in cereal yields in the mid-1980s (Harford et al. 2009:9). This decline in crop yields spurred government and NGOs to officialise CA, which culminated in a full-fledged farming system framework in the early 1990s. A case in point was the initiative called ‘Conservation Tillage for Sustainable Crop Production Systems’, instituted between 1988 and 1996. Its primary aim was to reduce soil loss and water runoff and improve crop yields (Marongwe et al. 2012:8).

Subsequently, in 1998, an international workshop dubbed ‘Conservation Tillage for Sustainable Agriculture’ was held in Harare to exchange knowledge on CA and to enhance its adoption at national and regional levels. The workshop involved local ministries and departments in the agricultural sector and high-profile stakeholders in agriculture such as FAO representatives, the German Agency for Technical Cooperation (GATC) and the Agricultural Research Council of South Africa, just to mention a few (Marongwe et al. 2012:8).

Apart from that, between 2003 and 2010, the local and international donor community complemented government efforts in promoting CA by adopting a transformed version of CA, which later was known as CF (Marongwe et al. 2012:9). Harford et al. (2009:3) noted that less than 40% of subsistence farmers do not have access to draught power. For that reason, NGOs promoting CF targeted poor and vulnerable subsistence farmers including those without access to draught power. Two major principles that underpinned the CF intervention were minimal soil disturbance and maintenance of soil cover with organic material. In line with these principles, NGOs embarked on outreach to promote the planting basin technique (the hand-hoe farming system) (Harford et al. 2009:3).

The point has to be made that while the government embraced CA, the majority of NGOs were advancing CF, primarily because their target groups were subsistence farmers who lacked access to draught power or other mechanised equipment (Mvumi et al. 2017:1631). A case in point was a local faith-based organisation called Foundation for Farming, which took it upon itself to train subsistence farmers in CF across different parts of the country. This training mainly focused on the hand-hoe-based farming system. As a result, there was an increased interest in CF, which attracted even government ministries, departments, NGOs and several stakeholders promoting CF, who reached an estimated 39 players including Christian CARE, CARE International, SAT, Action Aid, Dabane Trust and the Ministry of Education, to name a few (Marongwe et al. 2012:12). The planting basin technique was adaptable to the majority of subsistence farmers because of their lack of access to draught power and other cognate factors (Marongwe et al. 2012:9; Mvumi et al. 2017:1631).

This study focused on SAT, which was formed in 2007 and registered as a private voluntary organisation to promote CF in southern Africa and Zimbabwe in particular. In 2011, SAT’s name was changed to Sustainable Agriculture Technology (SAT 2014). According to SAT, provision of seed and fertiliser (inputs) in order to increase crop yields constituted a single component of food production intervention that could not achieve full impact. As a result, SAT incorporated training in land use (CF) and crop management to input provisions to increase farmers’ proficiency in food production (Hanyani-Mlambo et al. 2016:13; SAT 2014).

Ironically, SAT interventions in Zimbabwe occurred within the context of an agricultural extension service described by Hanyani-Mlambo et al. (2016) as:

… less effective and inefficient, limited prioritization by government, skills flight and institutional memory loss, inadequately trained and inexperienced frontline extension agents, fragmentation and confusion, duplication and the existence of parallel extension services, poor and/or non-existent research-extension-farmer linkages, adherence to top-down supply-driven and blanket recommendations … (p. 12)

Within this framework, this study evaluated the extent to which SAT support services were sustainable in Ward 13 of Bikita District.

## The setting and design

This study was conducted in Ward 13 of Bikita District. Ward 13 was selected for this study because of its proximity to the researcher and availability of SAT beneficiaries, with whom the researcher had prior contacts before conducting the study. Of the 31 wards in Bikita District, SAT covers six, namely, Wards 9, 10, 11, 12, 13 and 21. The impetus behind the selection of SAT was because, apart from training in CF, providing maize seed, sorghum seed, cowpea seed and chemical fertiliser, as is often the case with other cognate NGOs in the district, SAT assists in providing pesticides to deter pests that negatively affect maize crops (FAO 2008:4).

The study adopted a qualitative research design because of its strengths in the analysis and interpretation of any form of human story, attitudes or perceptions. Because the study sought to understand the views and attitudes of subsistence farmers, two prominent qualitative methods were instrumental, namely key respondent interviews and focus groups. Data were collected during the planting and weeding season (December 2009 and January 2010) in Ward 13 of Bikita District.

### Sampling method

A sample of 32 adults comprising 18 women and 14 men participated in this study. Key respondent interviews involving 11 adults – both male and female household heads – were conducted first, followed by focus group discussions. To select the first seven respondents for interviews, the researcher used purposive sampling. To select the other four respondents, existing networks were used, as beneficiaries of SAT linked the researcher with other like-minded respondents. The researcher stopped at 11 respondents after realising that he was getting the same information.

To achieve methodological triangulation, the researcher complemented interviews with focus group discussions. Based on proximity to each other, the researcher used snowball sampling to recruit three focus group respondents who were beneficiaries of SAT support packages from the three nearby villages. Before conducting focus group discussions, prior arrangements were made with gatekeepers and we scheduled discussions with all focus groups on a single day, which was the day selected for the fieldwork. Village heads were gatekeepers in all three focus groups. As a result, three focus groups, each with four women and three men, were administered in Ward 13. The six structured questions for both interviews and focus groups were as follows:

- Which crops do households grow?
- Which methods do they use to grow crops?
- What type of assistance does SAT provide to farmers?
- In your own view, do you think support from SAT is suitable for your type of soil?
- Using your own experience do you think farmers will become proficient in crop production after SAT has left?
- What must SAT do to improve its support services in future?

In running focus group discussions, the researcher worked with an adult male university undergraduate, whom the researcher carefully trained to moderate the focus groups. While the research assistant moderated the discussions, the researcher operated a voice recorder and observed the unfolding dynamics as well as writing notes.

On the age factor, the majority (14) of respondents were aged between 47 and 50 while seven were aged between 41 and 45. Eight were between 35 and 39 and three between 30 and 33. Regarding their level of education, 19 had attained secondary education while 13 had attained primary level. Respondents’ demographic characteristics indicated that they were economically active, as all are below 65 years, which is considered retirement age in Zimbabwe. Also, the attainment of primary and secondary education by all respondents is indicative of their level of literacy, which points to their capacity to comprehend the disseminated information during training in CF.

### Data analysis

Data analysis was ongoing during transcription. In analysing data, the researcher read the transcripts, coded the data and came up with themes. The identification of themes for discussion was made possible when the researcher devised a classification technique, as advised by Bless and Higson-Smith (1995), who recommended that data be categorised into themes. The researcher coded the data following the sequence of the interview guide.

### Ethical considerations

Lupane State University approved the interview protocols within the context of my post-graduate studies. After securing consent from the village heads and beneficiaries of the Sustainable Agriculture Trust. The researcher was at liberty to use a voice recorder to record interviews and focus groups, forming records on which he based his reports. To protect the dignity and confidentiality of all informants who participated in the study, the researcher used a coding system in which each respondent was represented by the common title ‘household head’. To distinguish each household head, gender and age of the household head were assigned. The coding system was devised in order to link direct quotes to specific respondents and to enable an audit trail of the data.

## Practical implications

### Crops grown by households in Bikita District

All (32) respondents were clear that their households predominantly grew maize. Other crops mentioned were groundnut, sorghum, rapoko, millet, wheat and cowpeas. Chikobvu et al. (2010:1) noted that maize is preferred because it has the potential to produce higher yields, it can be used in its green state and is not prone to grain-eating birds. In southern Africa, maize is not only grown in Zimbabwe but also in Angola, Malawi, Mozambique, South Africa and Zambia (Alliance for a Green Revolution in Africa 2014:138).

Regarding sorghum and other traditional crops such as millet and rapoko, the findings confirmed those of Chikobvu et al. (2010:1) that these crops are grown in areas that receive little rainfall such as Bikita District and that are located within Region IV. The diversification of crops in Bikita District is a welcome development. In Africa, crops such as maize, sorghum, cowpeas and millet are common. In the literature, crop diversity is explained as one of the factors that facilitates the increase of resilience among smallholder farmers in SSA (Alliance for a Green Revolution in Africa 2014:134; Gukurume 2013:89).

### Farming methods used to grow crops

All respondents (32) stated that they used ConvT as a farming method. Three-quarters (24) of respondents indicated that they used both CF and ConvT in order to see whether there was a difference in terms of yield. For example, one respondent said, ‘although [*the*] CF method conserves moisture, we still use ConvT because we are used to it’ (female household head from Ward 13, age 40). However, more than half (17) of respondents said that CF was better than ConvT in terms of the amount of inputs used, labour and yields. They stressed that CF has the potential to produce high yields with limited inputs and the capacity to improve soil that has been overworked. Another merit of CF was that less seed is used with the CF method because there is care in seed placement, and small quantities of seed cover a wide piece of land.

Other benefits of CF were reported by the FAO (2008), including that it:

Improves soil structure, water and air infiltration, reduce [*sic*] soil erosion and ultimately increases soil fertility…lands are kept weed free even during off-season so that residual moisture is not lost and weeds do not reach flowering stage to build up of [*sic*] the soil weed seed bank.(p. 4)

These results suggest that if improved land use is to be incorporated into the conventional input support services, subsistence farmers in Ward 13 of Bikita District are likely to experience improved crop yields, thereby achieving food security at household level.

Of particular note is that respondents (24) highlighted that with CF anyone, including children and women, can grow crops. These results indicate that CF performs superiorly because it counters traditional gender roles enforced by ConvT, in that CF can be practised by women and children, making it easier for all household heads to produce food for their families. This is true of a 45-year-old widow in Matopo District in Zimbabwe, who testified that she harvested 1.25 tonnes of millet from her piece of land (Dabane Trust 2009:5).

### The nature of assistance offered to subsistence farmers by Sustainable Agricultural Trust

All respondents (32) indicated that support from SAT involves the provision of input support and training in CF. On input support, respondents singled out that each household receives 10 kg of the ZM421 maize seed variety, 5 kg of sorghum seed, 2 kg of cowpea seed and 500 g of Bulldock pesticide. Apart from that, farmers are given chemical fertiliser: two 50 kg bags of compound D and ammonium nitrate per household. Moreover, results indicate that every respondent who participated in this study received inputs and training in CF. In the literature, it was demonstrated that inputs address farmers’ resource constraints but represent a single component of food production, which has some limitations in terms of achieving full impact. The incorporation of training in CF is seen as value addition to input support in that besides addressing soil infertility constraints, farmers are become proficient in improved land use and farming management techniques, which helps to boost crop yield (SAT 2014). The results confirmed previous studies stating that input support, training in CF and extension services, whether by government or donor organisations, help to reduce farmers’ vulnerability to hunger and malnutrition and to build food production capacity for smallholder farmers (FAO 2008; Freeman et al. 2004; Josserand & Darboe 2009).

### Suitability of assistance offered to subsistence farmers by Sustainable Agricultural Trust

All (32) respondents were clear that CF is an improved land use and farming management method. For them, training in CF is suitable because it came at their point of need, as their soils have been overworked. The suitability of CF planting basins was stressed by one respondent when she said, ‘basins capture rain and conserve moisture, and manure that is placed also conserve moisture’ (female household head from Ward 13, age 48). Respondents further pointed out that planting basins create ridges that prevent water runoff as well as conserve moisture. As results suggest, rainfall in CF is relevant because this technique is best suited to areas with low-rainfall and high temperatures such as Bikita (Chikobvu et al. 2010:1). Given that some subsistence farmers do not have cattle for draught powering, training in CF becomes versatile because farmers can use the hand-hoe technique and still produce improved yields. Over and above, subsistent farmers have adopted and employed CF as an alternative to ConvT, because the latter is responsible for the destruction of soil structure, leading to poor crop yields because of soil infertility and increasing environmental degradation.

Regarding the suitability of inputs, three-quarters (24) of respondents singled out the ZM421 maize and sorghum varieties as drought-resistant seeds that they received as support from SAT. Bikita District falls within Region IV, which receives low rainfall (Chikobvu et al. 2010:1). For that reason, the ZM421 maize variety was seen as suitable in Bikita because this maize variety does not require large amounts of rainfall. Furthermore, respondents indicated that the ZM421 maize seed variety is a short-seasoned variety, which means it matures early. One respondent stressed that ‘ZM421 matures early, that is, in 90 days as compared to R201, which requires 120 days to mature’ (female household head from Ward 13, age 48). These results confirm the *Africa Agriculture Status Report* (Alliance for a Green Revolution in Africa 2014:138), which states that the ZM421 maize variety is drought-resistant and is suitable in areas with low rainfall such as Bikita District.

Over and above that, respondents emphasised that the sorghum variety is also suitable because it can be used for 3 years as retained seed and still produce better yields. These results are in tandem with the literature, which states that subsistence farmers’ vulnerability to input constraints can potentially be reduced through input support, whether by government or NGOs (SAT 2014).

### Building livelihoods

All (32) respondents indicated that by offering assistance, SAT was building the livelihoods of households because farmers were getting inputs that they could not afford to purchase on their own. For example, one household head remarked that ‘some people do not have money to purchase inputs such as compound D, which is far beyond the reach of many smallholder farmers and some do not have cattle for draft power’ (female household head from Ward 13, age 36). Regarding the provision of chemical fertiliser by SAT, respondents pointed out that most subsistence farmers used to grow crops without applying fertiliser and their yields were not so lucrative but that after using fertiliser they witnessed an improvement in the yields. Respondents (24) further stated that they could now practise farming without draught power, a thing that they said was impossible before SAT assisted them. As Freeman et al. (2004) and FAO (2008) have found, the purpose of assistance is to restore household livelihoods in that farmers’ resource constraints are compensated for by support services. As a result, the SAT support programme in Bikita District can be regarded as relevant because it has met the beneficiaries at their points of need – most of them do not have cattle for draught power, they experience resource constraints and their land has been overworked. As such, training in CF and input support helps subsistence farmers to be self-reliant, improve soil fertility and crop yields, which is improved livelihood by another name.

### Sustainability of food support services offered by Sustainable Agricultural Trust

All (32) respondents pointed out that SAT had offered food production support services to assist farmers for a period of 2 years. Although the 2 years attributed to SAT can potentially create dependence attitudes among farmers, the time frame has a greater potential to build farmers’ capacity so that they can be proficient in CF practices (FAO 2008:4). However, because this study was concerned with sustainability, the accepted view was that farmers should be able to sustain themselves after SAT’s food production support programme had ended. Most (28) subsistence farmers’ optimism was based on the ZM421 maize variety, which is an open-pollinated variety, meaning that it can be planted for 3–4 years using retained seed and still produce better yields. They also stressed that because the ZM421 maize seed variety is an open-pollinated variety, when a farmer produces sufficient yields the variety can be planted for approximately 3 years and still produce good yields; each farmer can produce his or her own seeds for those years, thereby addressing input constraints.

Respondents further stated that crop seed such as cowpeas and sorghum could be replanted because they could convert part of the yield into seed for the next farming season. This suggests that support services by SAT, that are aimed at improving crop yields, would have been achieved in Ward 13 of Bikita District because farmers were able to continue with farming, even after SAT’s exit, using retained seed. Three-quarters (24) of respondents indicated that after harvest, surplus could be sold to raise cash to purchase other inputs, while a quarter (8) were of the view that organic manure could be used in place of fertiliser. For example, one household head was quoted as saying, ‘I can use the training that I got and sustain myself because I now know the importance of CF’ (male household head from Ward 13, age 48). The respondent’s comment implies that the farmer had become proficient in CF in crop production.

Furthermore, respondents (17) stated that even when SAT was gone, they would be left with knowledge about the creation of basins, seed planting, control of weeds and fertiliser application.

Sustainable Agriculture Trust’s food production support programme took 2 years (FAO 2008:4) in Ward 13 of Bikita District; such a timeframe can assist farmers to become very proficient in crop production, which can potentially be translated into sustainable land use and crop yields. Additionally, the maize, sorghum and cowpea seed varieties, which can be planted for 3 years using retained seed, help to confirm that farmers can continue to sustain themselves after SAT’s exit. On a positive note, this study was conducted 2 years after SAT had ceased to give support to subsistence farmers. As such, subsistence farmers practising CF for 2 years after SAT’s exit validated the fact that subsistence farmers had gained proficiency in crop production, thus reducing their donor dependence on food production.

The issue of food production sustainability is accompanied by the language that SAT officials use during training sessions. Because language is a medium of communication, it has positive or negative influence in terms of empowering subsistence farmers in Bikita District. In that regard, SAT has prioritised use of Shona, an indigenous language, during training sessions. The implications are that if farmers can comprehend the necessary information and apply the knowledge gained in their circumstances, improved land use and management can be achieved, resulting in higher yields. Accordingly, the maize, sorghum and cowpea seed varieties, which subsistence farmers planted, using retained seed for 2 years after SAT’s exit, and still got better yields, strongly support the sustainability of the work started by SAT in Ward 13 of Bikita District.

Results on the sustainability of support services to subsistence farmers are in contrast with studies by Chitongo (2013:53) in Ward 8 of Bikita District that showed that not every CF intervention was successful. His results refute the notion that, after adopting CF, food insecurity challenges faced by subsistence farmers can be mitigated. Similarly, studies by Mafongoya (n.d.:3) in Ward 21 of Bikita District showed low adoption of CF and pervasive resistance to CF by subsistence farmers. The case of Ward 13 of Bikita District showed that subsistence farmers there had adopted CF, which contrasts with Ward 8 of the same district, in which people were inimical to CF.

Overall, these studies suggest that not every intervention can be successful. These failures in Ward 8 of Bikita should be viewed as lessons learnt, but the adoption of CF in Ward 13 offers promise that training in CF and input support can improve farmers’ food production proficiencies.

## Recommendations to improve Sustainable Agricultural Trust support services in future

All (32) respondents stated that SAT should put up demonstration plots in every recipient village. Hanyani-Mlambo et al. (2016:8) noted that demonstration plots are ‘a key strategy for information dissemination and a platform for learning for farmers’. Respondents (20) further stated that existing demonstration sites were too far from beneficiaries’ places of residence, which made it difficult for casual site visits. Another recommendation was that, resources permitting, SAT should not limit support services to households without draught power, as every household should be equipped with knowledge in CF practice and access inputs. In addition, respondents suggested that in future SAT should provide and distribute T-shirts and hats to lead farmers after harvest in order to motivate other farmers in the village.

Regarding inputs, three-quarters (24) suggested that SAT should increase the quantities of maize seed currently offered to farmers from 10 kg to 25 kg per farmer. More than half (17) of respondents suggested that SAT should provide sugar bean, groundnut and oil seeds. A quarter (8) said they preferred groundnut seed to sorghum seed.

### Limitations of the study

One of the key limitations of this study is that it is based on data from a small number (32) of subsistence farmers in Ward 13. As mentioned before, the study is not intended to be a solitary undertaking but adds its voice to the limited number of studies on the sustainability of food production support services to subsistence farmers.

## Conclusion

The study evaluated the sustainability of good production support services offered by SAT to subsistence farmers. Support services offered to Ward 13 of Bikita District demonstrated the promise of the sustainability of SAT’s food production support services to subsistence farmers, especially poor and vulnerable household heads with constrained resources. The case of Ward 13 carries the implication that household food production challenges can be mitigated if actors such as NGOs complement government efforts through the provision of training in CF, input provision and other extension services to subsistence farmers. While in this study the sustainability of CF is believed to be basic to food production in Ward 13, the concern over its viability elsewhere would require a closer inspection of individual wards’ circumstances. To this end, it should be stated for the record that the maize, sorghum and cowpea seed varieties, which can be planted for 3–4 years using retained seed and still get better yields, strongly support the sustainability of food production support services offered by SAT to subsistence farmers in Ward 13 of Bikita District.
